# Vitamin K Antagonist–Induced Calciphylaxis in a Dialysis Patient With Antiphospholipid Syndrome: A Multidisciplinary Case Report

**DOI:** 10.1155/carm/8244011

**Published:** 2026-08-17

**Authors:** Rafat A. A. Atawneh, Ahmed Algharib, Rüdiger Möller

**Affiliations:** ^1^ Department of Vascular Surgery, Euregio-Klinik, Nordhorn, Germany

**Keywords:** antiphospholipid syndrome, calcific uremic arteriolopathy, calciphylaxis, end-stage renal disease, phenprocoumon, vitamin K antagonists

## Abstract

Calciphylaxis, or calcific uremic arteriolopathy (CUA), is a rare, life‐threatening syndrome characterized by microvascular calcification and cutaneous necrosis, predominantly affecting patients with end‐stage renal disease (ESRD) on maintenance dialysis, particularly those exposed to vitamin K antagonists (VKAs). We report the complex clinical course of a 35‐year‐old female patient with chronic kidney failure and triple‐positive antiphospholipid syndrome (APS) who presented with severe, painful bilateral thigh ulcerations. The patient had been receiving phenprocoumon for 2 years. Given her high‐risk profile for thrombosis, managing cutaneous necrosis while maintaining necessary anticoagulation presented a profound clinical dilemma. Histopathological evaluation confirmed the diagnosis of calciphylaxis. VKA therapy was immediately discontinued and replaced with renal‐adjusted apixaban to manage her underlying APS as an individualized rescue strategy. A multimodal, sequential wound care regimen was initiated, incorporating serial surgical debridements, negative pressure wound therapy (NPWT), autologous platelet‐rich plasma (PRP) infiltration, and acellular fish skin grafting. Following intensive multidisciplinary intervention involving vascular surgery, nephrology, and dermatology, the progression of the disease was successfully halted. Significant wound contraction, stable granulation, and complete epithelialization were achieved without systemic complications. This case underscores that short‐term VKA exposure can act as a critical trigger for calciphylaxis in patients with multifactorial risk profiles and demonstrates that a tailored transition to novel oral anticoagulants combined with advanced wound therapies can achieve favorable outcomes in highly complex, refractory scenarios.

## 1. Introduction

Calciphylaxis, also clinically recognized as calcific uremic arteriolopathy (CUA), is a rare, devastating, and potentially fatal thrombohemorrhagic cutaneous syndrome that predominantly strikes patients afflicted with end‐stage renal disease (ESRD), particularly those undergoing maintenance hemodialysis or peritoneal dialysis therapies [1, 2]. At its histopathological core, the condition is characterized by progressive medial calcification and concentric intimal hyperplasia of small subcutaneous arteries and cutaneous arterioles [3]. This microvascular remodeling limits perfusion, ultimately triggering microvascular thrombosis, tissue ischemia, localized infarction, and the formation of excruciatingly painful ischemic ulcerations that possess a remarkably poor long‐term clinical prognosis [3].

The systemic development of CUA is heavily dictated by a metabolic milieu that shifts the homeostatic balance away from native vascular protection and directly toward active hydroxyapatite crystallization. Established clinical risk factors for this condition comprise female sex, morbid obesity, diabetes mellitus, severe secondary hyperparathyroidism, chronic inflammatory states, and prolonged therapeutic exposure to vitamin K antagonists (VKAs) such as warfarin or phenprocoumon [4, 5]. VKAs pose an exceptionally severe hazard to these highly susceptible patient cohorts because they explicitly inhibit the gamma‐glutamyl carboxylation and functional activation of matrix Gla protein (MGP) [4, 5]. Under physiological circumstances, fully carboxylated MGP serves as one of the body’s most potent, indispensable endogenous structural inhibitors of ectopic medial vascular calcification [5]. When this protective molecular pathway is pharmacologically suppressed by a VKA regimen, systemic calcification cascades run unchecked within the vulnerable microvasculature, even when standard serum calcium and phosphate biochemical metrics remain completely balanced.

While CUA pathways are increasingly documented within standard uremic populations, its structural manifestation in a young patient presenting with a concomitant diagnosis of triple‐positive antiphospholipid syndrome (APS) poses an extraordinary clinical and therapeutic paradox. Triple‐positive APS places the patient at an extreme, catastrophic baseline risk for lifelong arterial and venous thromboembolism, a state that strictly dictates aggressive, perpetual systemic anticoagulation. Because VKAs represent the historical standard of care for triple‐positive APS cohorts, yet directly drive the development of CUA via MGP suppression, managing these opposing entities creates a critical clinical dilemma. This multidisciplinary case report delineates the unique diagnostic challenges posed by this combination and provides an advanced clinical blueprint for a successful therapeutic escalation combining an immediate anticoagulant switch to direct oral anticoagulants (DOACs) with sequential, bioregenerative microwound scaffolds.

This case report was prepared in accordance with the CARE guidelines (Supporting File 1).

## 2. Clinical Presentation

A 35‐year‐old female patient presenting with independently managed, ambulatory status was referred to our vascular surgery department due to the rapid progression of agonizingly painful, necrotic cutaneous lesions located bilaterally on her thighs. The patient’s medical background was highly complex, characterized by dialysis‐dependent chronic kidney disease secondary to severe septic shock suffered in March 2023. Concurrently during that admission, she was diagnosed with triple‐positive APS, necessitating the initiation of systemic anticoagulation with the VKA phenprocoumon on April 6, 2023. Other metabolic cofactors included an active tobacco smoking history and Class II obesity (BMI 37 kg/m^2^).

The clinical timeline of her dermatological crisis began in January 2025, when she initially observed a localized, asymptomatic bluish skin discoloration across the anterior and lateral aspects of both thighs. Mistaking these early manifestations for benign bruising, she did not seek immediate medical evaluation. Over the following months, the lesions progressively evolved from indurated plaques into highly debilitating, nonhealing ulcerations. This decline prompted an initial outpatient assessment on June 8, 2025, which demonstrated severe cutaneous breakdown and led to her urgent acute inpatient hospital admission on June 20, 2025. At admission, a detailed physical examination revealed severe tissue destruction across both lower extremities. On the right thigh, a 5 × 4 × 3 cm moist, gangrenous ulceration was visible, characterized by a central black necrotic eschar and substantial peripheral erythema. The contralateral left thigh exhibited an even more extensive and deeply excavated 8 × 6 × 4 cm ulceration showing ragged borders, active sloughing, and extensive underlying subcutaneous adipose necrosis (Figure 1). Both wound beds were exquisitely sensitive, producing severe, continuous ischemic pain that significantly limited her mobility.

**FIGURE 1:**
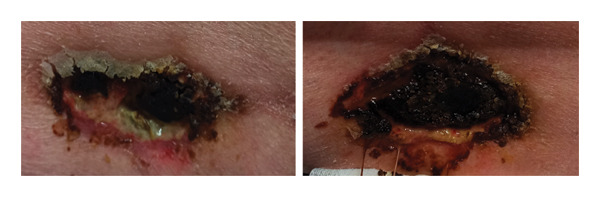
Clinical presentation upon admission showing bilateral thigh ulcerations.

Given the patient’s underlying triple‐positive APS, systemic necrotizing vasculitis and pyoderma gangrenosum were initially prioritized on the differential diagnosis list at admission. Due to this initial diagnostic ambiguity, empirical systemic corticosteroid therapy with prednisolone was initiated. To achieve diagnostic certainty, deep punch biopsies from the active, indurated margins of the bilateral thigh ulcerations were performed and submitted to the IPN Institut für Pathologie und Zytologie MVZ GmbH (Schüttorf/Leer, Germany). The subsequent histopathological analysis provided the definitive diagnosis, revealing the classic hallmarks of calciphylaxis. The biopsies demonstrated profound concentric tunica media calcification of the subcutaneous small‐ to medium‐sized arterioles, extensive intimal hyperplasia, and acute microvascular fibrin thrombi leading to complete luminal occlusion and secondary ischemic fat necrosis. Crucially, the histological evaluation showed no signs of vasculitis, granulomatous inflammation, or malignancy, thereby effectively ruling out the initial differentials (Figure 2). Consequently, the empirical corticosteroid therapy was systematically and rapidly tapered to prevent further impairment of wound healing. Regular monitoring of the patient’s serum biochemical parameters—including total calcium, inorganic phosphate, and intact parathyroid hormone (PTH)—revealed that these metrics remained consistently within normal physiological ranges throughout her entire maintenance dialysis regimen.

**FIGURE 2:**
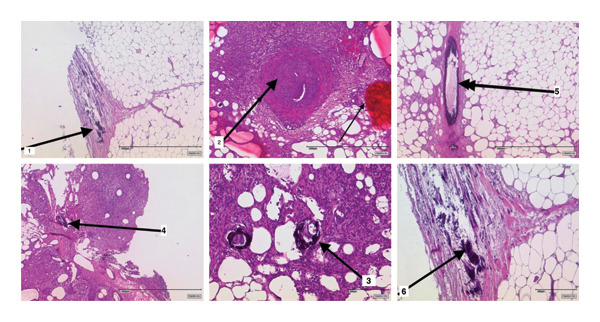
Histopathological evaluation of a deep skin punch biopsy from the ulcer margin (H&E staining). Panoramic views (40× total magnification) show classic diagnostic hallmarks of calciphylaxis: dense basophilic calcium deposits within fibrotic septa (Arrow 1), complete microvascular occlusion by an intraluminal fibrin thrombus accompanied by acute hemorrhage (Arrow 2), secondary ischemic lobular panniculitis (Arrow 4), and contiguous medial calcification in longitudinal section (Arrow 5). High‐power views (100× total magnification) delineate pathognomonic concentric medial calcification forming a distinct basophilic ring within an arteriolar wall (Arrow 3) and coarse crystalline calcium aggregates within the connective tissue matrix (Arrow 6). Image courtesy of IPN Institut für Pathologie und Zytologie MVZ GmbH.

Immediate interventions were enacted to eliminate systemic metabolic triggers and pharmacological risk factors. Oral calcium and vitamin D supplementations were promptly withdrawn. Systemic metamizole, initially used for pain management, was permanently discontinued after routine surveillance detected transient leukopenia, which normalized rapidly after cessation. Systemic antimicrobial therapy was withheld throughout the inpatient stay, as repeated clinical examinations and deep wound microbiological cultures demonstrated no secondary bacterial infection. Supportive biological maggot debridement therapy was proposed but declined by the patient.

To address the mandatory anticoagulation profile, phenprocoumon was immediately discontinued to halt VKA‐induced calcification pathways. However, complete withdrawal of antithrombotic protection was unfeasible given the catastrophic risk of systemic thromboembolism associated with her triple‐positive APS. Following intensive interdisciplinary consultation between the nephrology and hematology teams, the patient was transitioned to a renal‐adjusted DOAC regimen consisting of apixaban at a dose of 2.5 mg twice daily as an individualized rescue strategy. Concurrently, prednisolone therapy was systematically tapered, achieving complete discontinuation by August 2025.

Surgical management was structured around a strict chronological timeline aimed at initial macrostabilization followed by advanced biosynthetic tissue reconstruction. From June 20 to July 1, 2025, the patient underwent serial operating room procedures every 2 days, consisting of meticulous sharp mechanical debridements and radical necrosectomies targeting the extensive subcutaneous adipose necrosis across both thighs. Immediate stabilization of the freshly debrided wound beds was achieved through the application of negative pressure wound therapy (NPWT), maintained at a continuous negative pressure of −125 mmHg, which effectively controlled the ischemic exudate and stimulated localized granulation tissue. Following successful decontamination and the establishment of a clean, highly vascularized wound bed, the definitive bioregenerative phase was executed on July 11, 2025. First, a bilateral infiltration of autologous platelet‐rich plasma (PRP) was performed directly into the active, ischemic wound margins to deliver a highly concentrated bolus of native growth factors to trigger robust neoangiogenesis. Immediately following the biological infiltration, acellular fish skin grafts (Kerecis) were structurally fixed and implanted onto the deep, exposed wound beds of both thighs (Figure 3) to serve as an intact extracellular matrix scaffold for rapid reepithelialization. The patient was successfully discharged on July 14, 2025.

**FIGURE 3:**
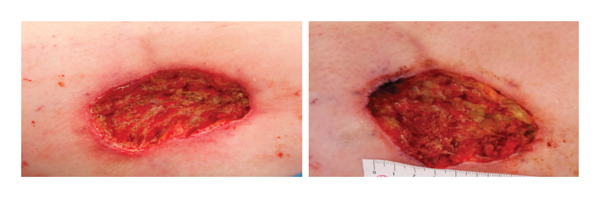
Intraoperative view following serial debridement and biological reconstruction.

Following the advanced surgical reconstruction, the patient entered a structured, multidisciplinary outpatient follow‐up program. During subsequent evaluations, she demonstrated exceptional clinical progress, including a rapid and marked reduction in excruciating ischemic pain, allowing for step‐by‐step physical rehabilitation and a successful return to baseline functional autonomy. From a systemic standpoint, the newly established therapeutic regimen demonstrated excellent tolerability. The transition to renal‐adjusted apixaban proved highly successful, with regular clinical and laboratory surveillance throughout the 6‐month follow‐up period confirming stable hematological parameters without any systemic bleeding complications or recurrent thromboembolic events. Furthermore, strict local wound assessments revealed a clean healing cascade with no clinical or microbiological signs of secondary infection. By December 2025, approximately 6 months after her initial acute admission, both extensive thigh ulcerations had achieved complete, stable closure with healthy, resilient scar tissue (Figure 4), and no recurrence was noted; the definitive diagnostic confirmation for this underlying pathology had been established by the baseline histopathological findings (Figure 2).

**FIGURE 4:**
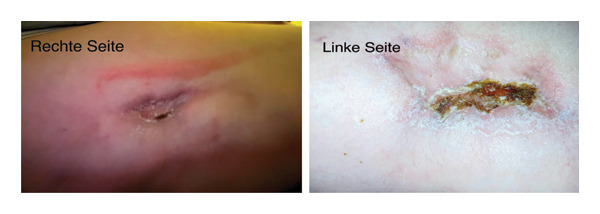
Long‐term clinical follow‐up 6 months postdischarge demonstrating complete, stable wound closure.

## 3. Discussion

This complex case heavily underscores a critical clinical doctrine: The presence of a completely normal calcium–phosphate product and normal PTH levels must never be used to exclude a diagnosis of calciphylaxis. It highlights that histopathological evaluation remains the indisputable gold standard for establishing a definitive diagnosis, especially when serum biomarkers are entirely inconspicuous [1, 3]. Furthermore, while the literature often associates this microvascular syndrome with decades of uremic exposure, this patient developed severe disease after only 2 years of VKA therapy with phenprocoumon. Rather than indicating that VKA exposure was the sole independent cause, this compressed timeline suggests that even short‐term VKA therapy can act as a critical compounding trigger that accelerates medial vascular calcification in a patient already highly vulnerable due to a multifactorial risk profile comprising ESRD, chronic inflammation, tobacco use, and morbid obesity.

Managing calciphylaxis within the clinical matrix of a young, dialysis‐dependent patient exhibiting triple‐positive APS introduces an extraordinary therapeutic paradox. International consensus guidelines for managing high‐risk triple‐positive APS mandate lifelong, stringent systemic anticoagulation, with VKAs serving as the uncompromised standard of care. This strict recommendation is driven by landmark clinical trial data demonstrating that DOACs are associated with inferior efficacy and significantly higher rates of arterial thrombotic failure in high‐risk APS cohorts. However, because VKAs directly drive the molecular pathogenesis of calciphylaxis through the systemic inactivation of MGP, their immediate and permanent cessation is paramount to halting fatal cutaneous ischemic progression [4, 5].

In our case, navigating this clinical dilemma required an innovative, carefully calculated intervention. While the patient exhibited multiple severe, synergistic risk factors for ectopic vascular calcification, the introduction of phenprocoumon for a duration of only 2 years likely acted as the definitive, compounding spark. Rather than implying that VKA exposure was the sole culprit, this clinical timeline underscores how even short‐term exposure can critically accelerate uremic calcification cascades, a concept conceptually rooted in early models of systemic calcification induction [6, 7].

Complete cessation of antithrombotic protection was entirely unfeasible due to the imminent threat of catastrophic APS‐related thrombosis, while low‐molecular‐weight heparin (LMWH) posed logistical, long‐term monitoring, and compliance challenges for ambulatory maintenance. Consequently, following an intensive interdisciplinary consultation, the patient was transitioned to renal‐adjusted apixaban at a dose of 2.5 mg twice daily. It is critical to acknowledge explicitly that DOACs are generally not preferred and are contraindicated in triple‐positive APS according to current guideline‐based evidence. However, this high‐risk decision represented an individualized rescue strategy in a highly complex clinical context where the standard treatment was actively driving a fatal dermatological condition. Emerging clinical and pharmacokinetic registries suggest that tailored low‐dose apixaban provides a viable, relatively safe alternative in advanced renal impairment [8]. This strategic switch successfully balanced the critical need to shield the patient from APS‐driven thrombotic events without perpetuating the microvascular calcification pathways accelerated by VKAs.

Additionally, while early literature occasionally described calciphylaxis as a predominantly nonulcerating condition in its prodromal stages [9], our case demonstrates the rapid, malignant progression from livedo‐like plaques to deep ischemic tissue destruction. When clinical suspicion is raised by painful, indurated, purpuric, or necrotic subcutaneous nodules, a prompt deep punch biopsy remains essential [1, 3]. Early histological confirmation is especially vital to differentiate calciphylaxis from mimicking pathologies like systemic necrotizing vasculitis or pyoderma gangrenosum. As observed in this case, the initial empirical use of systemic corticosteroids for suspected vasculitis was quickly recognized as nonindicated upon receiving histopathological clearance, allowing for a rapid steroid taper that preserved local tissue healing capacity and avoided further immunocompromise.

The major strengths of our approach lie in the execution of an immediate, targeted anticoagulant switch combined with a modern, sequential wound care protocol over traditional single‐modality strategies. Calciphylaxis ulcers are notoriously refractory to conventional dressings due to extensive microvascular thrombosis and local tissue hypoxia. By utilizing an aggressive, step‐by‐step strategy—commencing with the macrocleansing and exudate control of NPWT, followed by the microvascular angiogenic stimulation of autologous PRP [10], and finalizing with the structural matrix of acellular fish skin grafts [11]—we successfully bypassed the ischemic block. The application of autologous PRP delivered concentrated growth factors directly into the ischemic wound margins to enhance healing [10], while the biological wound dressing provided a robust extracellular matrix scaffold that minimized inflammatory degradation and promoted rapid reepithelialization [11]. This successful protocol offers a promising, reproducible roadmap for treating refractory uremic ulcers without relying on systemic sodium thiosulfate infusions, which carry an independent burden of severe metabolic acidosis and variable treatment outcomes [12].

Conversely, a notable limitation in this case was the patient’s delayed initial presentation, as she spent several months mistaking her early skin alterations for benign subcutaneous bruising. This delay reflects a widespread lack of clinical awareness regarding the early, nonulcerative symptoms of calciphylaxis among both patients and primary care providers. Enhancing clinical recognition of these early indurated plaques is vital, as timely intervention prior to frank ulceration and secondary tissue necrosis remains the most critical factor in reducing the high morbidity and mortality associated with this devastating syndrome [2].

## Funding

No external funding was received for this study.

## Ethics Statement

Institutional ethics committee approval was not required for this study due to the strictly noninterventional and fully anonymized nature of the case reporting.

## Consent

The patient provided comprehensive, written informed consent for the anonymized publication of her clinical history, relevant laboratory data, and accompanying clinical images.

## Conflicts of Interest

The authors declare no conflicts of interest.

## Patient Perspective

Initially, the patient mistook the early cutaneous changes and indurated plaques for harmless subcutaneous bruises, which led to a delay in seeking specialized medical care. However, upon definitive diagnosis and admission to our department, she reported feeling thoroughly informed, medically secure, and well‐supported by the interdisciplinary team. She highly valued being actively involved in the shared clinical decision‐making process regarding her complex treatment regimen. Furthermore, she found the structured outpatient wound care program highly effective and supportive, which significantly alleviated the logistical and physical burden of her long‐term recovery.

## Supporting Information

Additional supporting information can be found online in the Supporting Information section.

## Supporting information


**Supporting Information** Supporting File 1: CARE checklist. The CARE guidelines checklist for this case report has been completed and is provided as supporting information.

## Data Availability

No additional datasets were generated or analyzed during the current study. Further clinical or historical information regarding this case is available from the corresponding author upon reasonable request.
