# Supplementary material for: Vitamin K Antagonist–Induced Calciphylaxis in a Dialysis Patient With Antiphospholipid Syndrome: A Multidisciplinary Case Report
Source: Case Rep Med. 2026 Aug 17;2026:8244011. doi: 10.1155/carm/8244011 (PMC13478789; doi:10.1155/carm/8244011)
Supplement: Supplementary file 1 [file CARM-2026-8244011-s001.docx]

**CARE Checklist**

**Item Checklist item description Reported on Line**

| **Topic** |  | **Reported on Line** |
| --- | --- | --- |

| **Title** |  | **2-3** |
| --- | --- | --- |
| **Key Words** |  | **4-5** |
| **Abstract** | **3a** Introduction  **3b** Main symptoms  **3c** The main diagnoses, therapeutic interventions, and outcomes  **3d**Conclusion—What is the main “take-away” lesson(s) from this case? | **16-19**  **18-24**  **25-34**  **35-38** |
| **Introduction** |  | **39-74** |
| **Patient Information** | **5a** De-identified patient specific information  **5b** Primary concerns and symptoms of the patient  **5c** Medical, family, and psycho-social history including relevant genetic information  **5d** Relevant past interventions with outcomes | **75-78**  **79-81**  **82-83**  **84-85** |
| **Clinical Findings** |  | **86-104** |
| **Timeline** |  | **105** |
| **Diagnostic Assessment** | **8a** Diagnostic testing (such as PE, laboratory testing, imaging, surveys)  **8b** Diagnostic challenges (such as access to testing, financial, or cultural)  **8c** Diagnosis (including other diagnoses considered)  **8d** Prognosis (such as staging in oncology) where applicable | **106-143** |
| **Therapeutic Intervention** | **9a** Types of therapeutic intervention (such as pharmacologic, surgical, preventive, self-care)  **9b** Administration of therapeutic intervention (such as dosage, strength, duration)  **9c** Changes in therapeutic intervention (with rationale) | **144-216** |
| **Follow-up and**  **Outcomes** | **10a** Clinician and patient-assessed outcomes (if available)  **10b** Important follow-up diagnostic and other test results  **10c** Intervention adherence and tolerability (How was this assessed?)  **10d** Adverse and unanticipated events | **217-240** |
| **Discussion** | **11a** A scientific discussion of the strengths AND limitations associated with this case report  **11b** Discussion of the relevant medical literature with references  **11c**The scientific rationale for any conclusions (including assessment of possible causes)  **11d** The primary “take-away” lessons of this case report (without references) in a one paragraph conclusion | **241-310** |
| **Patient Perspective** |  | **311-320** |
| **Informed Consent** |  | **321-324** |
